# Decreased Perifoveal Sensitivity Detected by Microperimetry in Patients Using Hydroxychloroquine and without Visual Field and Fundoscopic Anomalies

**DOI:** 10.1155/2015/437271

**Published:** 2015-03-12

**Authors:** A. Molina-Martín, D. P. Piñero, R. J. Pérez-Cambrodí

**Affiliations:** ^1^Clínica Optométrica, Fundació Lluis Alcanyis, Universitat de Valencia, Calle de la Guardia Civil 22, 46020 Valencia, Spain; ^2^Oftalmar, Hospital Internacional Medimar, Edificio de Especialidades, 03016 Alicante, Spain; ^3^Departamento de Óptica, Anatomía y Farmacología, Universidad de Alicante, Carretera de San Vicente del Raspeig s/n, 03690 Alicante, Spain

## Abstract

*Purpose*. To evaluate the usefulness of microperimetry in the early detection of the ocular anomalies associated with the use of hydroxychloroquine. *Methods*. Prospective comparative case series study comprising 14 healthy eyes of 7 patients (group A) and 14 eyes of 7 patients under treatment with hydroxychloroquine for the treatment of rheumatologic diseases and without fundoscopic or perimetric anomalies (group B). A comprehensive ophthalmological examination including microperimetry (MP) and spectral-domain optical coherence tomography was performed in both groups. *Results*. No significant differences were found in mean MP foveal sensitivity between groups (*P* = 0.18). However, mean MP overall sensitivity was significantly higher in group A (29.05 ± 0.57 dB versus group B, 26.05 ± 2.75 dB; *P* < 0.001). Significantly higher sensitivity values were obtained in group A in comparison to group B for the three eccentric loci evaluated (*P* < 0.001). *Conclusion*. Microperimetry seems to be a useful tool for the early detection of retinal damage in patients treated with hydroxychloroquine.

## 1. Introduction

Hydroxychloroquine (HCQ) is the hydroxylated form of chloroquine (CQ), an antimalarial drug used by rheumatologists for the treatment of various systemic diseases including rheumatoid arthritis (RA) or systemic lupus erythematosus (SLE). The potential ocular toxicity of HCQ [1] was well known and was described first by Shearer and Dubois in 1967 [2]. The ocular side effects are less frequent and severe with HCQ compared with CQ and include asthenopia, paracentral scotoma, and color vision defects [3]. If those symptoms are overlooked, early degeneration of the retinal pigmentary epithelium (RPE) induced by HCQ can progress to an irreversible central visual loss. However, there is no agreement in how to establish a routine protocol for the early diagnosis of the HCQ retinopathy [4]. Likewise, new more sensitive techniques are needed to detect early changes and minimize visual loss. Microperimetry (MP) has demonstrated a wide potential for prediction, detection, and treatment monitoring in macular diseases [5]. This paper aims to evaluate the usefulness of this technique in the early diagnosis of HCQ maculopathy in asymptomatic patients with no fundoscopy findings or perimetric losses.

## 2. Materials and Methods

### 2.1. Patients

This prospective comparative case series study included a total of 28 eyes of 14 patients with ages ranging from 21 to 62 years. Patients with any type of active ocular disease or previous eye surgery were excluded. Two groups of eyes were differentiated: group A included 14 healthy eyes of 7 patients randomly selected and group B included 14 consecutive eyes of 7 patients using HCQ at least 1 year before the examination (mean exposure 12.33 ± 16.79 years (range 1 to 34 years)) and without fundoscopic or visual field alterations using a 10:2 threshold strategy (Humphrey Field Analyzer, Carl Zeiss, Germany). After the perimetric examination, mean deviation (MD) and mean standard deviation (MSD) were calculated. MD indicates the mean difference between the normal expected retinal sensitivity in terms of age and visual acuity and the measured patient sensitivity. Likewise, MSD evaluates the changes of the visual field pattern compared to the ideally expected ones in terms of patient age. Thus, a high MSD implies a significant focal visual field loss and a high probability of scotoma. All patients were given a written informed consent to participate in this study, which was approved by the local ethics committee. All the procedures followed the tenets of the Declaration of Helsinki.

### 2.2. Examination Protocol

A comprehensive ophthalmological examination was performed in all patients, including manifest refraction, corrected distance visual acuity (CDVA), slit-lamp examination, intraocular pressure (IOP), fundoscopy, MP, and spectral-domain optical coherence tomography (SD-OCT). MP was performed with the microperimeter MAIA (Centervue, Padova, Italy), which integrates the mechanism of the scanning laser ophthalmoscope (SLO) with the static perimetry. The mechanism of observation is an infrared superluminescent diode of wavelength 830 nm (Laser Class I, 60825-1 IEC: 2007) which provides images of high quality even with pupil diameters up to 2.5 mm. The maximum level of light of the perimetry is predetermined by the source of laser until levels of 318.47 cd/m^2^ and appears in ranges of attenuation from 0 to 36 dB in 1 dB steps. The background luminance is 1.27 cd/m^2^. A Goldmann-type size III stimulus is used in presentations of 200 milliseconds of duration.

**Figure 1:**
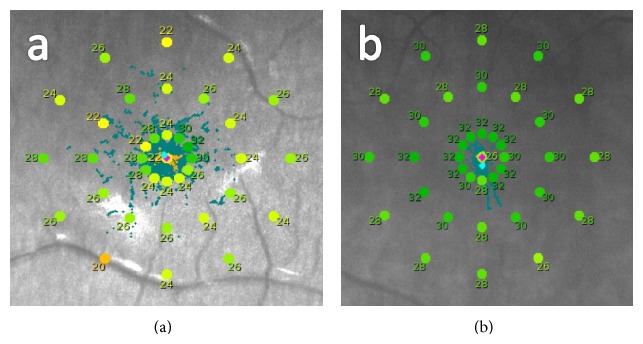
Examples of sensitivity maps obtained with the MAIA MP system: (a) sensitivity map of a patient under treatment with HCQ (group B); (b) sensitivity map of a healthy patient (group A).

The measures of sensitivity with the microperimeter were obtained using the option “Expert Exam,” consisting of the application of a 4-2 staircase strategy of threshold in a static exam. This examination allowed us to obtain the value of the threshold in each point in 1 dB steps. The type of predefined grid used for the perimetry consisted of 37 projection positions distributed in three concentric circles around the center placed at 1°, 2°, and 5° positions, containing 12 points each. After the examination, the results of sensitivity in the different positions evaluated and the average threshold (AT) were plotted and compared with a normative database provided by the instrument in order to classify the case as normal or not (Figures 1(a) and 1(b)). MAIA microperimeter also provides more specific information about the patient's fixation pattern obtained from the raw data, such as the bivariate contour ellipse area (BCEA) that represents the area of the ellipse that better fits the fixation points recorded during the measurement and that is obtained after estimating the major and minor axes of such ellipse. The area was estimated in square degrees considering 63% and 95% of points.

### 2.3. Statistical Analysis

Data analysis was performed using the software SPSS for Windows version 19.0 (SPSS Inc., Chicago, USA). Normality of data samples was evaluated by means of the Kolmogorov-Smirnov and the Shapiro-Wilk tests. When parametric analysis was possible, Student's *t*-test for paired data was used for comparisons between groups. When parametric analysis was not possible, the Wilcoxon rank sum test was applied to assess the significance of such differences. Differences were considered to be statistically significant when the associated *P* value was <0.05.

## 3. Results

The clinical characteristics of patients included in the sample are summarized in Table 1, represented by the mean values ± SD (range). Age, refractive error, CDVA, and retinal thickness data are provided for both study groups. No significant differences between groups in age (*P* = 0.603), refractive error (*P* = 0.352), or CDVA (*P* = 0.99) were detected. The analysis of OCT results showed no topographic changes and no significant differences between groups in central retinal thickness (*P* = 0.24), mean retinal thickness (*P* = 0.887), or retinal volume (*P* = 0.843).

Automated perimetric examination using a 10:2 threshold strategy resulted in a MD of −2.26 ± 0.91 (range −3.33 to −0.47) and a MSD of 1.19 ± 0.25 (range 0.79 to 1.54).

Mean MP foveal sensitivity was 26.86 ± 1.75 dB (range 24 to 29 dB) and 25.43 ± 2.65 dB (range 21 to 28 dB) in groups A and B, respectively. This difference was not statistically significant (*P* = 0.18). Mean MP overall sensitivity was significantly higher in group A (29.05 ± 0.57 dB, range 27.9 to 29.8 dB) compared to group B (26.05 ± 2.75 dB, range 20.4 to 29 dB) (*P* < 0.001).

The mean MP threshold values obtained for each ring were calculated for each patient in each group. These outcomes are summarized in Table 2. Significantly higher values were obtained in group A in comparison to group B for the three different eccentricities evaluated (2°, 6°, and 10°, *P* < 0.001). Likewise, significantly higher values of MP perifoveal sensitivity were found in group A compared to group B (28.04 ± 2.67 versus 25.29 ± 2.78 dB, *P* < 0.01).

The analysis of the fixation pattern showed no significant differences (*P* = 0.069) between groups A and B. Specifically, 95% of BCEA was 2.42 ± 3.48^°2^ (range 0.40 to 9.70^°2^) and 2.44 ± 2.71^°2^ (range 0.50 to 10.30^°2^) in groups A and B, respectively.

## 4. Discussion

Although uncommon, the incidence of CQ maculopathy is estimated between 1 and 6% and HCQ maculopathy below 1% [6]. HCQ toxicity mechanism is not yet fully understood, but Rodriguez-Padilla et al. reported an early damage of the ganglion cells and their photoreceptors in the perifoveal region [7]. The main risk factors for this toxicity include age, the cumulative dose [8], length of treatment longer than 5 years [9], and renal or liver disease that may increase the blood levels of these drugs. Although toxicity seems to be independent of daily dose and dose/kg ratio, this remains unclear. For this reason, it has been suggested not to exceed a daily dose of 400 mg for HCQ or of 250 mg for CQ [10]. Nevertheless, there is high variability in the cumulative doses that lead to toxic retinopathy [11] and the damage may appear even in those patients with a low systemic risk profile. Also, a high risk of progression of the retinal damage has been suggested despite the cessation of the treatment [12] and a “point of no return” for macular toxicity [13]. Thus, an individual and weight-adapted dosing has been proposed to minimize the incidence of retinal damage [14].

Currently, there is no “gold-standard” protocol of examination to confirm that there is ocular toxicity previous to the onset of an irreversible damage. Screening recommendations vary widely throughout the available scientific literature [15]. An exhaustive ocular examination is essential to establish a baseline profile in a patient who is going to be treated with HCQ and also to confirm if there is damage in an already treated patient. It should include a full medical history, VA with the best refractive correction, anterior segment biomicroscopy to detect cornea verticillata, and dilated fundoscopy to carefully evaluate the macula, where early signs of bull's eye maculopathy might be present in a long-term treated patient. Those changes are preceded by a loss of parafoveal sensitivity diagnosed by means of visual field examination (VFE). The recommended strategy is an automated threshold with a white 10-2 pattern. Other more sensitive but less accessible testing modes include multifocal electroretinography (mfERG) that allows the determination of local cone and bipolar cell activity at the posterior pole, fundus autofluorescence (FAF) imaging that may reveal the early deposition of debris in the outer segments of photoreceptors, and SD-OCT that may detect early thinning of the retinal layers in paracentral areas [10]. Nevertheless, some doubts still arise regarding these techniques. There is still not enough scientific evidence of the usefulness of FAF in the early diagnosis of HCQ maculopathy [16], and mfERG and SD-OCT failed to identify a significant number of cases of antimalarial retinal toxicity [17] and thus cannot be considered as gold-standard techniques to identify CQ and HCQ maculopathy.

Recently, the development of MP has allowed clinicians to early identify a reduced paracentral retinal sensitivity in those patients exposed to CQ and HCQ. Angi et al. reported a single case report showing a decreased sensitivity in an asymptomatic 60-year-old woman who had been receiving 3 mg CQ for 17 years for treating a severe RA. The patient showed a granular appearance of the macula but CDVA of 1.0 (decimal scale). Using the MP-1 microperimeter (Nidek Inc., Italy), these authors found a dense scotoma within the central 12°. The authors highlighted the usefulness of this technique in the follow-up as the tracking system allows the examination of exactly the same retinal points each time [18]. Martínez-Costa et al. conducted a controlled cross-sectional study in a sample of 209 patients taking HCQ or CQ to compare the microperimetric findings to 204 controls using the MAIA microperimeter. They found a significant depression in retinal sensitivity values in cases versus control subjects suggesting the usefulness of MP for the early detection of retinal toxicity [19]. Recently, these results have been corroborated by Jivrajka et al. in 16 patients under a long-term HCQ treatment and without clinical findings by means of mfERG, FAF, or SD-OCT techniques. They found a significant reduction of retinal sensitivity compared to controls [20]. According to our results, MP is a perimetric technique that provides enough information about retinal sensitivity and can be considered as a screening clinical procedure for the early detection of retinal damage in patients treated with antimalarial drugs. It is especially useful for detecting retinal alterations that cannot be detected in the fundoscopic examination or by OCT. Specifically, the obtained results, in absence of significant damage in the 10:2 threshold strategy perimetry observed in the study group of our sample, are the distinguishing feature compared to the previous research and permit paving the way for MP to become the gold-standard diagnostic technique in the early diagnosis of HCQ maculopathy. Future studies evaluating larger samples of eyes would be desirable to corroborate these results and to analyse if parafoveal changes in retinal sensitivity begin immediately after the beginning of the treatment.

## Figures and Tables

**Table 1:** Clinical characteristics of the two groups of the analyzed sample.

	Control group	HCQ group
Age (years)	38.86 ± 12.56 (21 to 62)	40.86 ± 10.49 (31 to 62)

Sphere (D)	−0.20 ± 1.02 (−2.25 to 1.25)	−0.52 ± 1.61 (−4.25 to 0.75)

Cylinder (D)	−0.52 ± 0.83 (−2.50 to 0.00)	−0.52 ± 0.95 (−2.75 to 0.00)

Spherical equivalent (D)	−0.46 ± 0.94 (−2.25 to 1.25)	−0.78 ± 2.04 (−5.63 to 0.38)

CDVA (decimal scale)	1.00 ± 0.00 (1.00 to 1.00)	1.05 ± 0.16 (0.90 to 1.50)

Central thickness (*μ*m)	210.3 ± 42.3 (181 to 306)	209.7 ± 18.2 (176 to 232)

Average thickness (*μ*m)	272.0 ± 14.6 (258.9 to 301.3)	269.2 ± 14.7 (249.3 to 286.6)

Volume (mm^3^)	7.69 ± 0.41 (7.32 to 8.52)	7.61 ± 0.41 (7.05 to 8.10)

CDVA: corrected distance visual acuity; D: diopters; *μ*m: micrometers.

**Table 2:** Mean retinal sensitivity in each ring of the grid presented for the two groups of the analyzed sample for both groups.

	Control group	HCQ group	*P* value
Ring 1° (dB)	30.20 ± 1.02 (28.67 to 32.17)	27.36 ± 2.64 (21.17 to 30)	<0.01

Ring 2° (dB)	29.16 ± 0.50 (28.17 to 29.83)	26.96 ± 2.65 (20.67 to 29.50)	<0.01

Ring 5° (dB)	28.04 ± 0.67 (26.50 to 28.92)	25.29 ± 2.78 (19.33 to 28)	<0.01

dB: decibel.
